# Genetic predictors of acute toxicities related to radiation therapy following lumpectomy for breast cancer: a case-series study

**DOI:** 10.1186/bcr1526

**Published:** 2006-07-18

**Authors:** Christine B Ambrosone, Chunqiao Tian, Jiyoung Ahn, Silke Kropp, Irmgard Helmbold, Dietrich von Fournier, Wulf Haase, Marie Luise Sautter-Bihl, Frederik Wenz, Jenny Chang-Claude

**Affiliations:** 1 Department of Epidemiology, Division of Cancer Prevention and Population Science, Roswell Park Cancer Institute, Buffalo, New York, USA; 2 German Cancer Research Center, Division of Clinical Epidemiology, Heidelberg, Germany; 3 Department of Gynecological Radiology, Heidelberg University Hospital, Heidelberg, Germany; 4 Clinic for Radiotherapy and Radiooncology, St. Vincentius-Kliniken Karlsruhe, Karlsruhe, Germany; 5 Clinic for Radiotherapy, Karlsruhe Hospital GmbH, Karlsruhe, Germany; 6 Department of Radiation Oncology, Universitätsklinikum Mannheim, Mannheim, Germany

## Abstract

**Introduction:**

The cytotoxic effects of radiation therapy are mediated primarily through increased formation of hydroxyl radicals and reactive oxygen species, which can damage cells, proteins and DNA; the glutathione S-transferases (GSTs) function to protect against oxidative stress. We hypothesized that polymorphisms encoding reduced or absent activity in the GSTs might result in greater risk for radiation-associated toxicity.

**Methods:**

Women receiving therapy in radiation units in Germany following lumpectomy for breast cancer (1998–2001) provided a blood sample and completed an epidemiological questionnaire (*n *= 446). Genotypes were determined using Sequonom MALDI-TOF (*GSTA1*, *GSTP1*) and Masscode (*GSTM1*, *GSTT1*). Biologically effective radiotherapy dose (BED) was calculated, accounting for differences in fractionation and overall treatment time. Side effects considered were grade 2c and above, as classified using the modified Common Toxicity Criteria. Predictors of toxicity were modelled using Cox regression models in relation to BED, with adjustment for treating clinic, photon field, beam energy and boost method, and potential confounding variables.

**Results:**

Low activity *GSTP1 *genotypes were associated with a greater than twofold increase in risk for acute skin toxicities (adjusted hazard ratio 2.28, 95% confidence interval 1.04–4.99). No associations were noted for the other GST genotypes.

**Conclusion:**

These data indicate that *GSTP1 *plays an important role in protecting normal cells from damage associated with radiation therapy. Studies examining the effects of *GSTP1 *polymorphisms on toxicity, recurrence and survival will further inform individualized therapeutics based on genotypes.

## Introduction

Breast conserving surgery followed by radiation therapy has been shown to be as effective as mastectomy in preventing breast cancer mortality [1], and use of this approach in women with localized disease is common. Although breast irradiation is fairly well tolerated, treatment is often associated with short term side effects, including skin erythema and irritation, as well as medium-term and long-term side effects of breast edema, pain, fibrosis, and telangiectasia [2]. Side effects of radiation therapy have been shown to have an impact on quality of life, with women undergoing therapy exhibiting a decrease in overall quality of life during treatment, measured by a modified Breast Cancer Chemotherapy Questionnaire, compared with those who did not have breast irradiation following surgery [3]. Breast irritation and pain were greatest among women who had longer term therapy.

In breast cancer, effort has been made to identify factors other than radiation dose that may predispose to damage to normal tissue in the targeting of residual cancer cells with radiation therapy following lumpectomy. In previous investigations, systolic blood pressure [4], breast size [5], and age [6] were associated with acute skin reactions in breast cancer patients. In a prospective cohort of women with breast cancer receiving radiation therapy following lumpectomy, with carefully calculated dose and monitored acute skin toxicity, we noted that higher body mass index (BMI), but not DNA repair capacity, was associated with acute side effects [7].

These factors explain only a portion of the variability in acute side effects of radiation therapy, and efforts have been made to identify genetic factors that could make normal cells more sensitive to contralateral damage with treatment. Radiation therapy exerts its cytotoxic effects through direct action (imparting energy to electrons that damage DNA directly) and indirect action [8] by producing reactive oxygen species (ROS), including superoxide radicals, hydrogen peroxide, and hydroxyl radicals [9,10]. Thus, it is plausible that genetic variability in protection from oxidative stress could influence susceptibility to acute side effects in women receiving radiation therapy. Glutathione-associated metabolism is a major mechanism for cellular protection against ROS and their toxic products, and glutathione *S*-transferases (GSTs) α, μ, π, and θ are active in detoxifying organic epoxides, hydroperoxides, and unsaturated aldehydes, including reactive bases and lipid peroxides produced by reactive oxidant damage to DNA and lipids, respectively [11,12]. *GSTM1 *and *GSTT1 *both contain gene deletions, resulting in no enzymatic activity for that isozyme. The GSTA1*A and GSTA1*B genetic polymorphism (RS3957357), containing three linked base substitutions in the promoter at positions -567, -69, and -52, results in differential expression [13], with lower transcriptional activation of the GSTA1*B (variant) than of the GSTA1*A (common) allele *in vitro *[14]. The *GSTP1 *isoleucine^105^valine substitution is also associated with reduced activity [15].

Reduced detoxification of ROS and their products resulting from polymorphisms in genes encoding GSTs could be responsible for greater acute toxicities among women receiving radiation therapy. In a prospective study of genetic and nongenetic factors that could influence the effects of radiation therapy on normal tissue, we evaluated the role of polymorphisms in GST genes *GSTM1, GSTT1, GSTA1 *and *GSTP1 *in predicting acute side effects.

## Materials and methods

For this study of genetic and nongenetic predictors of skin toxicity associated with radiation therapy, an unselected cohort of women with breast cancer was recruited from several radiotherapy units in Germany from 1998 to 2001. As previously described [7,16], women were eligible if they were receiving primary radiation therapy following breast conserving surgery and had not previously received chemotherapy. Participating radiotherapy units included those of the Women's Clinic in Heidelberg, the University Hospital of Mannheim, the St. Vincentius Clinic in Karlsruhe, and the City Hospital of Karlsruhe. Women were all Caucasian and there were no age restrictions; informed consent was obtained from all patients. Of the 506 patients recruited, eight were excluded because of unsatisfactory data collection, and 20 patients withdrew their consent during the period of data collection, for a total of 478 patients for whom data were available for the present analysis. The study was approved by the Ethical Committee of the University of Heidelberg, the Institutional Review Board for Roswell Park Cancer Institute, and the US Army Medical Research and Materiel Command Human Subjects Research Review Board. Participants were asked to complete a questionnaire with epidemiologic information on demographic and lifestyle factors, medical history, and family history of breast cancer. Permission was also requested to incorporate clinical data (tumor characteristics and therapy regimen) into the research database, and for collection of a blood specimen before the initiation of radiation therapy.

As previously described [16], all patients received standard breast irradiation treatments, including computed tomography based planning, simulation, verification, and quality assurance, with conformal tangential irradiation with lateral and medial wedge fields. All regimens included irradiation of the entire breast. At three units, women received either 50 Gy given in five 2.0 Gy fractions per week or 50.4 Gy given in five 1.8 Gy fractions per week, followed by boost radiation from 6 to 25 Gy. At the fourth radiation department, 56 Gy of whole breast irradiation were applied in five 2.0 Gy fractions per week without a boost. To account for differences in fractionation and overall treatment time, the biologically effective radiation therapy dose (BED) was calculated using the following formula:

,          

where n is the number of fractions, d is the fraction size, α/β is the ratio for acute skin reactions (10 Gy), γ/α is the time factor (0.7 Gy/day), T is the overall treatment time, and T_0 _is the starting time for compensatory proliferation of 21 days [17]. The average BED for radiotherapy by censoring was 54.0 ± 4.8 Gy, with a range from 35.5 to 64.5 Gy.

Side effects were carefully monitored and recorded by treating physicians, and toxicities were documented four times during the study. These included before the beginning of therapy, and at cumulative doses of 36–42 Gy, 44–50 Gy, and 60–66 Gy (end of radiation therapy). Acute side effects were classified using a modified system based on the Common Toxicity Criteria of the Cancer Therapy Evaluation Program (National Institutes of Health, 1998 [18]. For this analysis, we included only acute side effects of grade 2c and above, which is categorized by at least one moist desquamation or interruption of radiotherapy due to toxicity [19]. Of the patients who provided a blood specimen (*n *= 446), 77 women presented with toxicity of grade greater than 2c.

Genomic DNA from pretreatment blood samples was extracted from lymphocytes using the QIAamp DNA Blood Midi Kit (Qiagen, Hilden, Germany), in accordance with the manufacturer's instructions. All DNA preparations were stored at 4°C until use. Genotyping for polymorphisms in *GSTA1 *and *GSTP1 *was performed by BioServe Biotechnologies (Laurel, MD, USA) using Sequenom's (San Diego, CA, USA) high-throughput matrix-assisted laser desorption/ionization time-of-flight (MALDI-TOF) mass spectrometry.

PCR was performed using the following primers: for *GSTA1*, 5'-ACGTTGGATGTTAAACGCTGTCACCGTCCT-3' and 5'-ACGTTGGATGGAGTGGCTTTTCCCTAACTTG-3'; and for *GSTP1*, 5'-ACGTTGGATGTGGTGGACATGGTGAATGAC-3' and 5'-ACGTTGGATGCAACCCTGGTGCAGATGCTC-3'. A multiplex PCR technique that detects homozygous deletions of *GSTM1 *and *GSTT1 *was used, including primers for the β-globin gene as an internal control, with an annealing temperature of 62°C [20]. For *GSTM1*, primers 5'-GAACTCCCTGAAAAGCTAAAGC-3' and 5'-GTTGGGCTCAAA TAT ACGGTGG-3' were used; and for *GSTT1*, primers T1 (5'-TTCCTTACTGGTCCTCACATCTC) and T2 (5'-TCACCGGATCATGGCCAGCA) were used. The absence of amplified *GSTM1 *or *GSTT1 *product (in the presence of the albumin PCR product) indicated the respective null genotype for each. All genotyping results were reviewed manually for quality control. Controls for genotype and two 'no template' controls were included on each plate. In addition, 170 sets of blinded controls (8%) were distributed throughout the plates for quality control purposes. Because there were differential failures in genotyping by gene, numbers vary somewhat in the tables and columns.

The associations of toxicity with personal or treatment characteristics (age, BMI, skin sensitivity, skin disease, smoking status, alcohol use, radiotherapy of lymph nodes and hormone therapy), as well as genotypes (*GSTM1, GSTT1, GSTA1*, and *GSTP1*), were analyzed using the Cox model. Univariate analysis for each factor was conducted initially to estimate crude associations. To be included in the model for multivariate analysis, the variable had to be potentially significant (*P *< 0.30 with the likelihood ratio test) by univariate analysis. The relative hazard (with 95% confidence interval) for toxicity compared with a reference group was also estimated as an indication of the strength of the possible association. Because the treatment algorithm was slightly different across four clinics, and photon-field and boost method are considered the additional factors affecting toxicity to biologic dose, the Cox regression analysis was conducted adjusted for photon and boost methods, as well as stratified by clinic. This strategy was implemented to control for dose and other treatment factors. The toxicity-free (without toxicity of grade 2c or greater) probability along with BED was estimated using the Kaplan-Meier method. All the analyses were performed using SAS 9.1 (SAS Institute, Cary, NC, USA) software.

## Results

**Figure 1:**
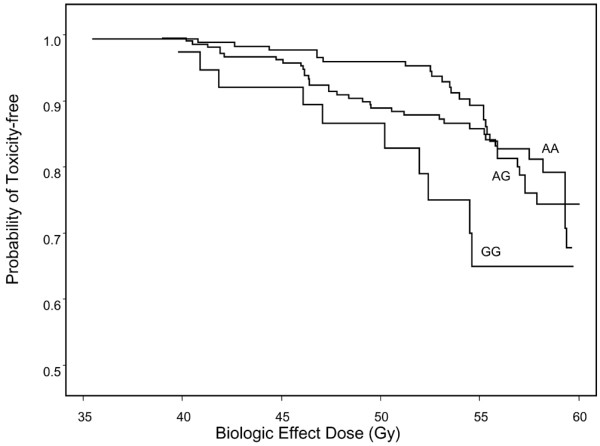
Probability of toxicity-free effects. Kaplan-Meier curve for the probability of toxicity-free effects (without Common Toxicity Criteria grade 2c toxicity or greater) by *GSTP1 *genotypes.

As previously reported [7] and shown in Table 1, of all demographic and epidemiologic factors evaluated, only BMI was associated with a greater risk for experiencing severe skin toxicities following radiation therapy, although there was an association of borderline significance with alcohol consumption (yes/no). When evaluating the potential effects of genotypes associated with reduced or absent activity for the GSTs, we found that *GSTP1 *GG genotypes, associated with reduced enzyme activity, were significantly associated with acute side effects of radiation therapy (Figure 1). When models were adjusted for clinic, photon field, beam energy, boost method, BMI, smoking status, alcohol consumption, and hormone therapy (Table 2), there was more than a twofold increase in hazard of toxicity among women with *GSTP1 *GG genotypes (adjusted hazard ratio 2.28, 95% confidence interval 1.04–4.99), in comparison with women with common AA genotypes. Although there was a slight increase in risk in women with heterozygous genotypes, suggesting a dose-response effect, the association was not statistically significant (hazard ratio 1.38, 95% confidence interval 0.83–2.30), although there was a significant gene dosage effect (*P *= 0.04). There were no associations noted for *GSTM1*, *GSTT1*, or *GSTA1*. When 'at-risk' alleles were combined (Table 3), and women with two or more alleles related to reduced or absent activity were contrasted with those with 0 or 1 low activity alleles, no cumulative effects were noted.

## Discussion

In this analysis of potential relationships between genotypes related to reduced protection from ROS and severe side effects of radiation therapy following lumpectomy for breast cancer, we found that carrying the *GSTP1 *isoleucine^105^valine substitution (GG genotype) was associated with a more than twofold increase in risk for experiencing acute skin toxicities. Genotypes associated with reduced or absent activity for *GSTM1*, *GSTT1*, and *GSTA1 *were not associated with increased toxicity.

To our knowledge, there have been no evaluations of the role of genotypes associated with decreased GST activity and side effects experienced with radiation therapy. However, there have been a number of studies designed to address the effects of GST genotypes on survival and secondary malignancies after treatment for cancer, with several – although not all – studies finding an important role for *GSTP1 *polymorphisms in treatment outcomes. In a study of patients with Hodgkin's lymphoma [21], the low activity *GSTP1 *genotype was associated with better survival. In that population, patients were treated with a number of chemotherapeutic agents, so the mechanism is not specific to radiation-generated ROS. Similar relationships with *GSTP1 *were noted in a study of patients with esophageal cancer [22], with no effects on survival observed for *GSTM1 *and *GSTT1*. This patient population did include those who did not receive adjuvant therapy, and the survival effects were observed among both treated and untreated populations. However, in another study that measured GSTP1 expression in esophageal tissue among patients receiving chemoradiation, higher expression was associated with poorer survival [23], strengthening the hypothesis that reduced levels of GSTP1 are associated with greater cell kill. In an earlier study of women receiving treatment for breast cancer, we found that low activity *GSTP1 *genotypes were associated with better survival [24], and these results were replicated in the Shanghai Breast Cancer Study [25]. In our earlier study [24] the majority of women received chemotherapy plus radiation therapy, and in the Yang study only chemotherapy was administered as adjuvant therapy. To our knowledge, one study assessed genetic polymorphisms and susceptibility to radiotherapy-related malignancies, but only evaluated *GSTM1 *and *GSTT1*, finding nonsignificant elevated risk for second cancers among those with gene deletions [26].

Assessment of relationships between GST polymorphisms and survival following cancer treatment is based on the hypothesis that reduced GST activity would result in fewer ROS and therapeutic agent metabolites reaching tumor cells, where they could confer protection against damage to DNA, proteins, and lipids. Our investigation of toxicities associated with radiation therapy following lumpectomy is based on the premise that this same inability to block ROS might result in better tumor cell kill but also in concomitant damage to normal tissue in the breast, demonstrated by adverse skin toxicities. There is an absence of data regarding this issue, although earlier work did show that prevention of breast cancer relapse with radiation therapy was greatest among women with elevated levels of GST π protein in tumor tissue, supporting a role for GSTP1 in response to radiation therapy [27]. Because radiation results in generation of ROS and lipid peroxidation, the finding that nuclear GST π plays a role in the cellular sensitivity to oxidative stress caused by hydrogen peroxide through scavenging the formation of lipid-peroxide modified DNA [28] further support our findings.

Our confidence in the finding reported here, namely that low activity *GSTP1 *genotypes are associated with greater skin toxicity following radiation therapy, is bolstered by the design of the study and careful assessment of toxicity outcomes. This study was specifically designed and conducted to determine the role of genetic factors in skin toxicities, with *a priori *hypotheses established before implementation of the study. Each radiation dose was recorded, and cumulative radiation dose was calculated. Women were monitored and scored for skin toxicities according to cumulative dose time points, and were categorized according to the Common Toxicity Criteria of the National Institutes of Health.

In this same cohort, we previously evaluated the role of polymorphisms in DNA repair genes (*XPD*, *XRCC1*, and *APE*) in acute side effects of radiation [16]. No effects were noted among the total population, but when analyses were limited to women of normal weight, risk for side effects was greatly reduced among carriers of *APE1 *^148^Glu and *XRCC1 *^399^Gln alleles, with no reduced risk observed among overweight and obese women. We did not note an effect of BMI in our analyses (data not shown), but we did observe the associations between greater toxicity and low activity *GSTP1 *genotypes among the entire study group.

## Conclusion

In summary, in this carefully designed and conducted study of genetic predictors of acute side effects from radiation therapy following lumpectomy for breast cancer, we found that women with *GSTP1 *genotypes encoding lower activity were more likely to experience toxicity of grade 2c and above. These findings are consistent with several studies that found better survival among cancer patients with low activity *GSTP1 *alleles, implying that genotypes associated with lower GSTP1 activity allow reactive intermediates to interact with and damage both tumor and normal cells. Of course, therapeutic decisions regarding optimal dose must strike a balance between treatment efficacy in preventing cancer recurrence and reducing severe side effects. Studies that incorporate such measures (toxicities, recurrence, and survival) will help to clarify this issue and provide clinically relevant data on the utility of using genotypes for individualized therapeutics.

## Abbreviations

BED = biologically effective dose; BMI = body mass index; GST = glutathione *S*-transferase; PCR = polymerase chain reaction; ROS = reactive oxygen species.

## Competing interests

The authors declare that they have no competing interests.

## Authors' contributions

CA conceived the study and obtained grant funding, coordinated genotyping efforts, supervised data analysis, and drafted the manuscript. CT and JA participated in data management and statistical analysis, and in drafting the manuscript. SK and IH participated in the design of the original study, coordination of the original study, data collection and management, and in drafting the final manuscript. DVF, WH, MS-B, and FW participated in design of original study, designed assessment of exposure (radiation BED), were the principal investigators of the study at each site, and participated in drafting of final manuscript. JC-C designed and conducted the original study, co-designed the genetic study, coordinated all aspects of conduct of the study, and co-drafted manuscript. All authors read and approved the final manuscript.

## Figures and Tables

**Table 1:** Patient characteristics and skin toxicity (>2c) among women receiving radiation therapy following breast conserving surgery

Characteristics	Patients (*n*)	Toxicities (*n*)	HR^a ^(95% CI)
Age (years)			
≤50	62	11	1.0
>50 to 60	168	36	1.29 (0.65–2.57)
>60 to 70	152	23	0.84 (0.41–1.76)
>70	65	8	0.78 (0.31–1.97)
BMI (kg/m^2^)			
≤25	229	23	1.0
26–30	150	36	2.50 (1.45–4.30)
>30	68	19	3.30 (1.76–6.21)
Skin sensitivity			
Always sunburn	113	17	1.0
Sometimes sunburn	243	43	1.15 (0.65–2.04)
Never sunburn	80	16	1.49 (0.74–2.99)
Current smoking			
No	399	67	1.0
Yes	48	11	1.54 (0.79–3.01)
Alcohol consumption			
No	110	12	1.0
Yes	321	61	1.88 (1.00–3.53)
Radiotherapy of lymph nodes			
No	397	67	1.0
Yes	50	11	1.32 (0.66–2.63)
Hormone therapy			
No	68	9	1.0
Yes	379	69	1.51 (0.75–3.04)

^a^Hazard ratio adjusted for clinic, photon field, beam energy, and boost method; numbers vary because of missing data. BMI, body mass index; CI, confidence interval; HR, hazard ratio.

**Table 2:** Associations between polymorphisms in GST genes and skin toxicity (≥2c) following radiation therapy for breast cancer

Genotypes	Patients	Toxicities	HR^a ^(95% CI)
*GSTM1*			
Present	215	36	1.0
Absent	213	39	1.23 (0.74–2.03)
*GSTT1*			
Present	384	69	1.0
Absent	55	6	0.73 (0.29–1.86)
*GSTA1*			
GG	149	29	1.0
GA	194	35	1.09 (0.65–1.82)
AA	87	12	0.85 (0.43–1.71)
			*P *(trend) = 0.44
*GSTP1*			
AA	176	27	1.0
AG	213	39	1.38 (0.83–2.30)
GG	38	10	2.28 (1.04–4.99)
			*P *(trend) = 0.04

^a^Hazard ratio adjusted for clinic, photon field, beam energy, boost method, BMI, smoking status, alcohol consumption, and hormone therapy; numbers vary because of missing data. CI, confidence interval; GST, glutathione *S*-transferase; HR, hazard ratio.

**Table 3:** Effects of combined alleles encoding reduced detoxification on radiation-related toxicities

Number of alleles	Patients	Toxicities	HR^a ^(95% CI)
0 or 1	149	22	1.0
2	148	33	1.19 (0.61–2.382)
3 or 4	102	17	1.26 (0.72–2.21)

*P *trend = 0.67. CI, confidence interval; HR, hazard ratio.
